# Therapeutics of Pyrenol

**Published:** 1906-09

**Authors:** 


### Therapeutics of Pyrenol.

DR. F. WALTHER, of Leipzig, (*Therap. Neuheiten,* No. 3, 1906) reviews the reports on pyrenol published by Ewald's clinic, Redtenbacher's division, Schlesinger, Gruenfeld, Koehler, Silber, Burchard, Komor, Sternberg, Fasano, Maramaldi, Steiner and others. Pyrenol is chiefly expectorant, sedative, antipyretic and antirheumatic. It does not affect blood pressure and pulse, as almost all salicylates do; on the contrary, it is tonic and stimulant. Stomach and intestines are in no way injured even by its continued use; the appetite improves. All authors are agreed that it has no untoward effects of any kind.

Its most striking results are seen in asthma, where it produces material relief, even of dyspnea. Secretion is lessened and the attacks are rendered milder and often checked altogether. It is almost as valuable in pertussis, diminishing the number and the intensity of the spasms. It acts exceedingly well in croupous and catarrhal influenza, influenzal pneumonia, acute bronchitis and typhoid. Here its antipyretic action is supported by its expectorant and analgesic virtues.

Its content of salicylic acid, which is chemically combined to a readily soluble salt, makes it especially useful in articular and muscular rheumatism, gout and, in larger doses, in neuralgias. Finally, Walther states that pyrenol is used with benefit also in cardiac neuroses and subjective difficulties occasioned by cardiac affections. The average adult dose is 7½ to 15 grains t. i. d. In pneumonia and typhoid 7½ grains every three hours are given; children get one-half and infants one-fourth of this dose in cold water, milk, and other vehicles.

